# Absence of association of IFNL3/IL28B rs 12979860 and IFNL4 ss 469415590 polymorphisms with the neurological status of HTLV-1 Afro-Caribbean subjects in Martinique.

**DOI:** 10.1186/1742-4690-12-S1-P61

**Published:** 2015-08-28

**Authors:** Severine Jeannin, Jean-Marc Costa, Jean-Dominique Poveda, Gilda Belrose, Agnes Lezin, Andre Cabie, Raymond Cesaire, Stephane Olindo

**Affiliations:** 1 Service de Neurologie, Centre Hospitalier Universitaire de Martinique, Fort-de-France Cedex, Martinique; 2 Service de Viro-Immunologie, Centre Hospitalier Universitaire de Martinique, Fort-de-France Cedex, Martinique; 3 Service des maladies Infectieuses, Centre Hospitalier Universitaire de Martinique, Fort-de-France Cedex, Martinique; 4 Pasteur-CERBA, Cergy-Pontoise, France

## Background

The polymorphism of Interferon-lambda3/Interleukine28B (IFNL3/IL28B) rs 12979860 has been described as important in the development of HTLV-1-associated myelopathy/spastic paraparesis (HAM/TSP). Recently, the dinucleotide polymorphism, IFNL4 ss469415590 has been discovered and is in high linkage disequilibrium with rs12979860. In a transversal study, we aimed to examine the polymorphisms of these two nucleotides in our HTLV-1 Afro-Caribbean population.

## Methods

The frequencies of the CC, CT and TT genotypes of the single nucleotide rs12979860 and the frequencies of ΔG/ΔG, ΔG/TT and TT/TT genotypes of the dinucleotide ss469415590 are reported in the entire HTLV-1 group and compared between asymptomatic individuals and HAM/TSP patients.

## Results

In our 94 HTLV-1 subjects, frequencies of rs1299860 were CC, 13.3%; CT, 44.7%; TT, 42% and frequencies of ss469415590 genotypes were ΔG/ΔG, 45.7%; ΔG/TT, 42.6%; TT/TT, 11.7%. We found no significant difference in allele distribution in both studied nucleotide polymorphism between 53 asymptomatic carriers (60.7 years and 72% females) and 41 HAM/TSP patients (70 years and 80% females).

## Conclusion

In our population, the polymorphisms of IFNL3/IL28B rs12979860 and IFNL4 ss469415590 are not associated with HTLV-1 neurological phenotype status. Different genotypes target should be considered.

